# A Rare Case of Prostatic and Bilateral Renal Abscesses Caused by Community-acquired Methicillin-resistant Staphylococcus aureus Infection

**DOI:** 10.7759/cureus.7046

**Published:** 2020-02-19

**Authors:** Vrinda Vyas, Timothy Endy

**Affiliations:** 1 Internal Medicine, State University of New York (SUNY) Upstate Medical University, Syracuse, USA

**Keywords:** mrsa, prostatic abscess, staphylococcus aureus

## Abstract

Gram-negative bacilli are usually implicated in the formation of prostatic abscesses (PA) which is a rare complication of prostatitis. However, PA methicillin-resistant Staphylococcus aureus (MRSA) has emerged as a substantial cause of PA in recent years. Predisposing factors for MRSA prostatitis include immunocompromised states such as human immunodeficiency virus (HIV), uncontrolled diabetes, intravenous drug use (IVDU), urethral instrumentation, bladder outlet obstruction, preexisting prostatic disease, recent prostatic procedure, and chronic dialysis among others. MRSA PA should be promptly diagnosed and appropriately treated as delay in diagnosis can be detrimental. We present a case of a patient with a remote history of IVDU who developed simultaneous bilateral renal and PA caused by MRSA in the absence of MRSA bacteremia. Since our patient did not have the traditional risk factors for MRSA infection, we can argue that he was infected by the community-acquired strains of MRSA.

## Introduction

A prostatic abscess (PA) is an uncommon consequence of bacterial prostatitis. It is commonly a result of infection with Escherichia coli and other gram-negative bacteria of the Enterobacteriaceae family. Prostate abscess formation is noted only in 0.5% to 2.5% of patients presenting with acute bacterial prostatitis [1]. Staphylococcus aureus is rarely implicated as the causative organism in PA, but it is being increasingly reported, especially in patients with diabetes, immunocompromised state, and urinary tract abnormalities. A literature review identified 40 cases of Staphylococcal PA, of which 26 were methicillin-resistant [1].

We report a case of a 53-year-old patient with a history of injection drug use who presented with suprapubic tenderness, and was found to have bilateral renal and PA caused by methicillin-resistant Staphylococcus aureus (MRSA) in the absence of MRSA bacteremia. He underwent drainage of renal abscesses and required a prolonged antibiotic course.

We report this case to highlight that MRSA infection is increasingly being identified in unusual sites including the urinary tract and prostate. Also, the lack of a standardized diagnostic and therapeutic approach to PA complicates the treatment of this uncommon condition [2].

## Case presentation

**Figure 1:**
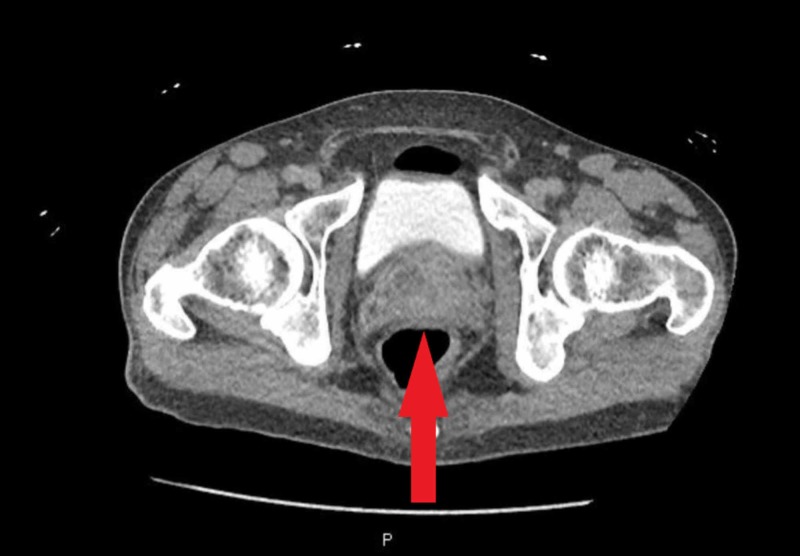
Prostate abscess extending into the seminal vesicles bilaterally seen on computed tomography scan with contrast

**Figure 2:**
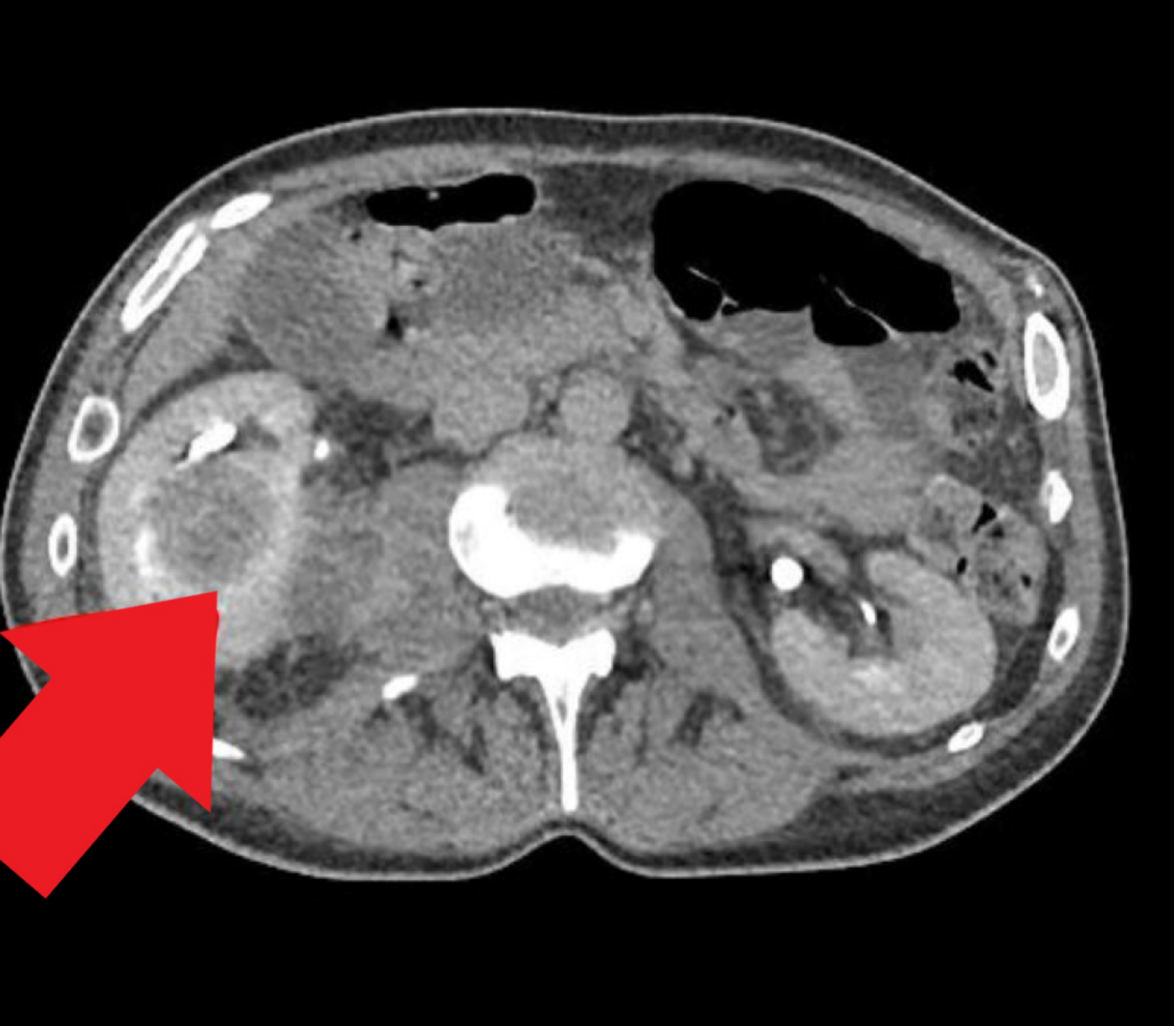
Right renal abscess on the computed tomography scan

**Figure 3:**
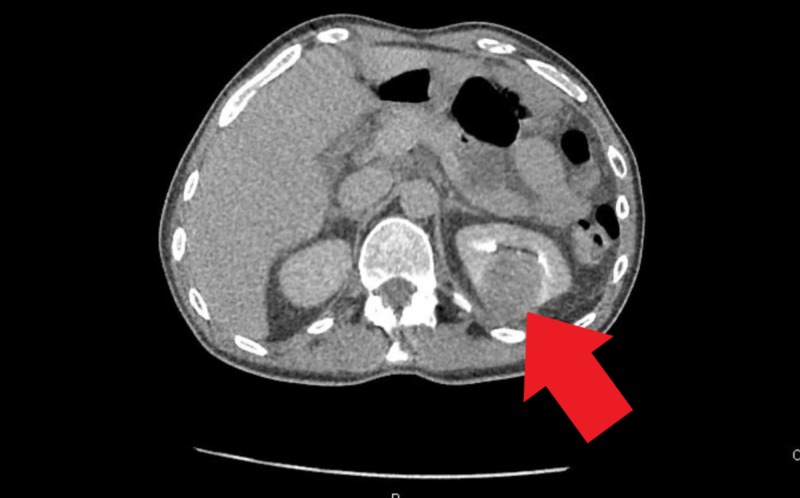
Left renal abscess as noted on the computed tomography scan

A 53-year-old man with a medical history significant for schizophrenia, hypertension, and intravenous drug use (IVDU) presented to the hospital with complaints of abdominal pain for one week with associated nausea, vomiting, diffuse myalgia, malaise and decreased appetite for one month. The patient also reported a chronic non-productive cough without hemoptysis and night sweats over the past one year. The patient reported that he stopped IVDU almost two years ago. On initial evaluation, the patient was afebrile, other vitals within limits. Physical examination revealed suprapubic tenderness, costovertebral angle tenderness and was otherwise unremarkable. Laboratory workup revealed leukocytosis (white blood cell (WBC) 12.6x103/uL) and transaminitis (alanine aminotransferase (ALT) 206/aspartate aminotransferase (AST) 166 IU/ml). Urinalysis revealed 42 WBCs/ HPF, 2+ leukocyte esterase, trace bacteriuria, urine culture was awaited. Computed tomography (CT) of the abdomen and pelvis with contrast revealed multiloculated abscesses in both the kidneys and a 5.7 cm multicystic abscess in the prostate extending into the bilateral seminal vesicles (Figures 1-3).

Blood cultures were obtained in the emergency department. The patient was then started on intravenous cefepime and vancomycin. Urology was consulted and the patient underwent drainage of the bilateral renal abscesses with bilateral nephrostomy tube placement by the interventional radiology (IR) team. IR deemed the patient to be at high risk for PA drainage and hence, it was deferred. In the meantime, urine cultures resulted positive for MRSA (40,000 colonies/ml). The purulent aspirate obtained during abscess drainage also grew MRSA. Surprisingly, both sets of blood cultures from admission were sterile. Transthoracic and transesophageal echocardiography did not show any evidence of infective endocarditis. Infectious disease (ID) was consulted for antibiotic recommendations; they recommended four weeks of vancomycin and sulfamethoxazole/trimethoprim double strength (800/160 mg). HIV and infective hepatitis screening were negative.

**Figure 4:**
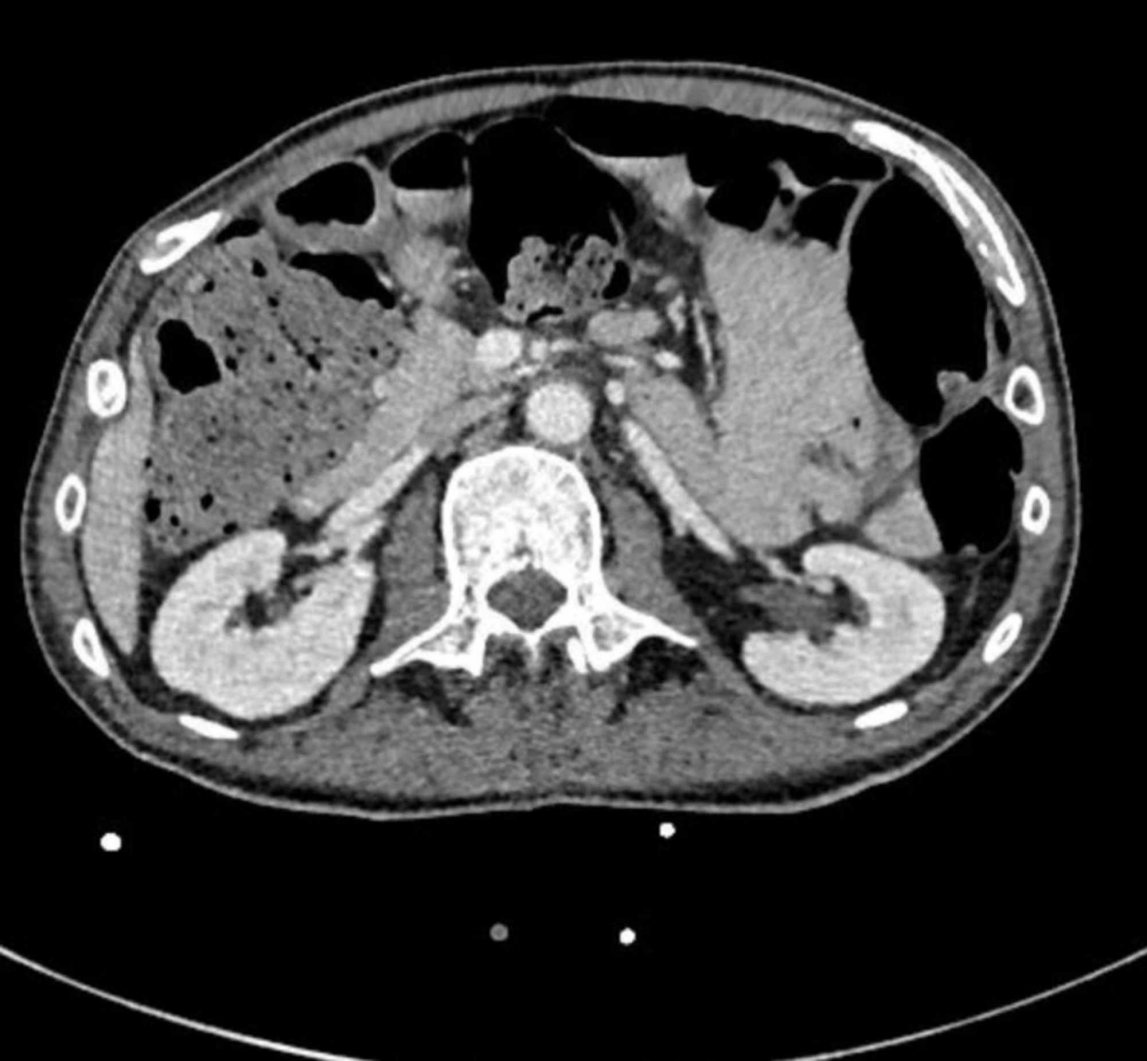
Resolution of bilateral renal abscesses

**Figure 5:**
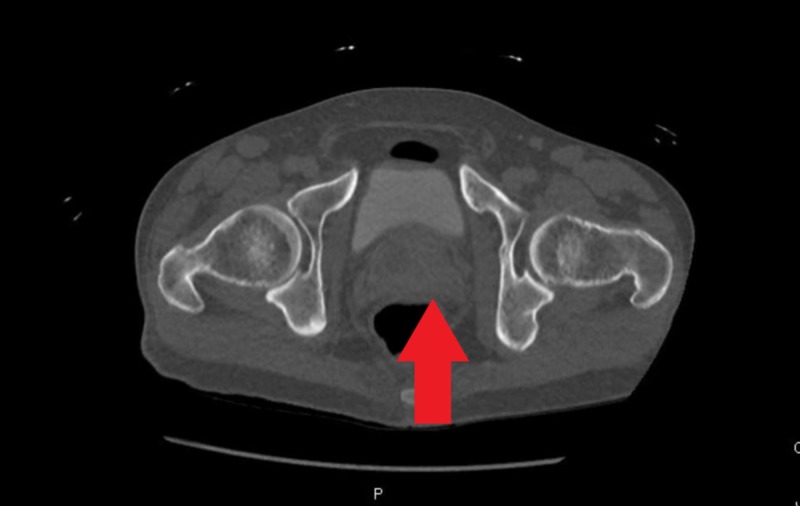
Resolution of prostatic abscess after antibiotic therapy but the prostate is still enlarged

A follow-up CT of abdomen and pelvis at the end of therapy to look for resolution of the abscesses was performed at four weeks, which showed interval decrease in bilateral renal abscesses, resolution of the PA but demonstrated a persistently enlarged prostate suspicious for either a benign or a malignant prostate pathology (Figures 4-5).

After reviewing the latest CT scan, urology recommended a prostate biopsy, but the patient declined it; also, the prostate-specific antigen (PSA) level was obtained, which was within normal limits for the patient’s age. ID recommended continuing sulfamethoxazole/trimethoprim double strength (800/160 mg) for two more weeks. Bilateral nephrostomy tubes were removed in the interim as they stopped draining purulent material consistent with the CT findings of decreased size of renal abscesses. The patient was discharged after five weeks of antibiotic therapy with instructions to continue one more week of sulfamethoxazole/trimethoprim as an outpatient. The patient reported complete resolution of symptoms at the time of discharge. The patient will follow up with urology in the clinic to reassess the needs for a prostate biopsy to exclude underlying primary prostate pathology. The patient will also follow up with ID specialists.

## Discussion

A PA is formed by a collection of purulent material within the prostate. It is a rare complication of acute bacterial prostatitis, occurring in about 0.%5 to 2.5% of patients presenting with prostatitis [1]. Risk factors for PA include instrumentation and urinary tract surgery, bladder voiding issues, recent prostate biopsy, IVDU, diabetes, and other immunocompromised states. Multiple mechanisms for the formation of a PA have been described, including urethral infection, instrumentation and injury to the prostatic urethra, reflux of infected urine into the prostate, and hematogenous spread [3].

Before the advent of modern antibiotic therapy, 75% of the PA were caused by Neisseria gonorrhea and the mortality rate was up to 30% [4]. The causative agents of PA have changed in recent years and now gonococcal PA has become rare while E. coli and other gram-negative bacteria have become the most implicated pathogens [3]. Staphylococcus aureus is a relatively rare cause of urinary tract infection (UTI) and prostatic infection, but it is being more frequently reported with a literature review identifying 40 cases of Staphylococcal PA, of which 26 were methicillin-resistant [1].

MRSA was initially discovered as a nosocomial pathogen affecting hospitals and other environments with high antibiotic use [5]. Nowadays, MRSA contributes to up to 40% of all infections caused by Staphylococcus aureus [6]. Since the late 1990s, multiple cases of MRSA infection have been reported in healthy individuals residing in a community setting, without any traditional risk factors for MRSA infection such as recent hospitalization, chronic stay in long term care facilities, the presence of a chronic indwelling device or catheter, etc. [7,8]. These cases of community-acquired MRSA (CA-MRSA) infection have been noted to causes 8%-20% of all Staphylococcus aureus infections. CA-MRSA usually involves the skin and soft tissue infections, but UTI and prostatitis caused by it is extremely rare [8].

Staphylococcus aureus bacteriuria is often considered to be a consequence of Staphylococcus aureus bacteremia with the primary source of infection located elsewhere in the body (e.g. endocarditis) [9]. Our patient’s presentation with MRSA bacteriuria in the absence of bacteremia and valvular vegetations, lack of immunosuppression, diabetes, urethral instrumentation is a rare occurrence. Isolation of Staphylococcus aureus from urine samples in the absence of bacteremia is therefore often considered to represent colonization of the urinary tract rather than a true UTI [9]. In certain patient populations, such as those in a long-term care facility (especially patients with urinary catheterization, prolonged antibiotic use), Staphylococcus aureus is a well-recognized pathogen causing UTI [9]. Our patient did not have the typical risk factors for a Staphylococcal UTI, and no other possible source of Staphylococcus infection was identified.

Our patient likely had a primary prostatic pathology which caused urinary retention; hence, we can postulate that he developed primary MRSA prostatitis complicated by a PA which caused an ascending MRSA UTI leading to renal and perinephric abscesses bilaterally.

PA present with varied clinical signs and symptoms, many of which overlap with UTI and acute bacterial prostatitis including fever, dysuria, increased urinary frequency, urinary retention, perineal pain, back pain, and hematuria. This makes the diagnosis difficult in the absence of imaging studies. There is usually fluctuance and pain on prostate palpation on digital rectal examination, which is characteristic of prostate abscess [10].

If there is clinical suspicion for a PA, imaging with transrectal ultrasound (TRUS), CT scan should be performed promptly. TRUS is the imaging of choice because it allows for simultaneous needle aspiration if a drainable pus collection is noted [11].

When not adequately treated or when treatment is delayed, the PA has the potential to cause sepsis and even death [12]. Early diagnosis and effective therapy are thus required. However, there is no standardized diagnostic and therapeutic approach for the management of prostate abscess caused by MRSA [1]. Empiric treatment with broad-spectrum antibiotics effective against E. coli and MRSA are the treatment of choice for patients with PA. Antibiotics should then be tailored according to culture and sensitivity reports of blood, urine, or tissue samples. Surgical therapies like perineal incision and transurethral resection are usually performed along with antibiotic therapy.

Further research and reporting of Staphylococcus aureus PA are required for understanding the pathogenesis and treatment of this uncommon condition [1].

## Conclusions

A PA is an underdiagnosed pathology and one due to MRSA is exceedingly rare. With the increasing incidence of MRSA PA, it is evident that MRSA is spreading its influence into the gram-negative territory of infection. We report this case to highlight the rare occurrence of MRSA PA with simultaneous bilateral renal abscesses in the absence of MRSA bacteremia.
